# Parental Expectations, Anxiety, and Health Behaviors in Routine Childhood Vaccination: Associations with Child Pain and Symptom Experiences

**DOI:** 10.1016/j.jpedcp.2026.200227

**Published:** 2026-07-01

**Authors:** Brian A. Jorge, Sterre van der Ziel, Elske Hogendoorn, Michel J. van Vliet, Judith G.M. Rosmalen

**Affiliations:** 1 Department of Psychiatry, University of Groningen, University Medical Centre Groningen, Groningen, The Netherlands; 2 Beatrix Children's Hospital, University of Groningen, University Medical Centre Groningen, Groningen, The Netherlands; 3 Department of Internal Medicine, University of Groningen, University Medical Centre Groningen, Groningen, The Netherlands

**Keywords:** Expectations, health behaviors, early childhood, parents, symptom report

## Abstract

**Objectives:**

In young children, somatic symptom reporting occurs through parents. Parental cognitions can directly influence the reported symptoms in their child after vaccination. Limited research exists on factors affecting parental symptom reporting. The current study examined whether parental state anxiety and side effect expectations before routine vaccinations are associated with parent reports of pain and symptoms after vaccinations. In addition, we explored health behaviors in relation to symptom expectations and observed symptoms.

**Study design:**

We used data from routine vaccinations of TRAILS-NEXT cohort participants aged 3, 11-12, and 45-48 months (n = 295, 300, and 167, respectively). Parents completed prevaccination questionnaires on anxiety and side effect expectations. They rated child pain directly after vaccination, and reported on observed symptoms in the child and parental health behaviors 1 week after vaccination. Associations were analyzed using multivariable linear regression.

**Results:**

Higher parental anxiety before vaccination was associated with increased reported pain ratings at 3 months. Anxiety and side effect expectations were not significantly associated with the overall symptoms experienced in the week after vaccination. When exploring specific symptoms, parents tended to observe the symptoms they expected at 3 months, but not at older ages. Parental health behaviors were primarily associated with child age and observed symptom burden, rather than with parental expectations.

**Conclusions:**

These findings suggest that parental anxiety affects parental pain report in young children post vaccination, potentially shaping both symptom experience and parental symptom-related behavior. Addressing and managing parental anxiety may reduce perceived vaccination pain severity in parents of young children.

## Highlights

• Parental anxiety predicted higher acute pain reports at 3 months.
• Expectations did not predict total symptoms 1 week post vaccination.
• Specific expected symptoms matched observations only at 3 months.
• Temperature checks and medication rose with observed symptom burden.
• Health behaviors related more to observed symptoms than expectations.

Children in many countries receive vaccines against common infections. Studies show that vaccination side effects are mostly mild and short term, although symptoms such as local pain, redness, swelling, headache, or fever do occur.^1^, ^2^, ^3^, ^4^, ^5^ Parents are informed about possible side effects, which shape expectations, emotions, and cognitions about postvaccination symptoms. Expectations about pain and side effects can influence symptom perception and pain experience.^6^^,^^7^ Young children often cannot accurately communicate their symptom experience. As such, symptom reporting occurs through parents, whose emotions and cognitions can directly affect reported pain and symptoms.^8^

Research shows parental pain estimates often relate to anxiety and worries during procedures, with frequent incongruence between parent and child ratings.^9^, ^10^, ^11^ One study found prevaccination expectations strongly predicted side effect reporting days and weeks later. Here, concerns about vaccine safety and parental anxiety increased symptom reporting, partly due to heightened monitoring.^12^

Parental help-seeking after vaccination side effects is also influenced by emotions and cognitions. Parents seek help more often when their child is distressed or when they lack prior vaccination experience. Social norms, such as avoiding unnecessary doctor visits, also play a role.^13^ Negative attitudes toward vaccination and prevaccination anxiety were found to increase the likelihood of seeking medical attention for side effects.^14^^,^^15^

Research on parental expectations of vaccination side effects remains scarce. Only 1 study examined expectations and anxiety in relation to side effect reporting in a small and selective sample.^12^ Whether specific expectations predict parental reports of acute vaccination pain and vaccination side effects, and subsequent behaviors, is unknown. Research on the nocebo effect, that is, when negative expectations cause individuals to experience more symptoms, suggests that higher expectations can increase attention to symptoms.^16^ Parents may report expected symptoms more often, but this has not been fully investigated. Similarly, the influence of expectations and anxiety on acute pain reports during vaccination is unclear, with limited evidence in children older than 24 months of age.^17^ How expectations affect parental responses, such as seeking advice or giving medication, also remains unexplored.

This study aimed to examine parental cognitions, emotions, and health behaviors surrounding routine vaccination in young children. First, we examined whether parental expectations and parental anxiety, measured before vaccination, were each individually associated with parent's reports of acute pain immediately after vaccination. Second, we assessed how these factors related to symptom reports during the week after vaccination. We hypothesized that higher parental expectations and higher parental anxiety would each be associated with greater reports of acute pain and more postvaccination symptoms. Third, we explored whether there was an association between symptoms that parents expected before vaccination and those they observed in the week after vaccination. Finally, we examined health behaviors after vaccination in relation to the expected likelihood of symptoms and reported symptom burden.

## Methods

The current study used data from the Tracking Adolescents’ Individual Lives Survey (TRAILS)-NEXT cohort, an intergenerational spin-off including the children of participants of the TRAILS cohort.^18^ The TRAILS-NEXT study was approved by the Central Committee on Research Involving Human Subjects (NL47782.042.14; amendment NL47782.042.14). The collection of data during routine vaccinations, started in October 2020, was approved by the Central Committee on Research Involving Human Subjects (NL47782.042.14).

### Study Population

TRAILS includes 2 cohorts: a population cohort (n = 2230) following preadolescents since 2001 to study mental health and social development,^19^ and since 2004 a clinical cohort (n = 543) of children referred to a child psychiatric outpatient clinic before age 11. Pregnant TRAILS participants are invited to join TRAILS-NEXT, which examines how parental development and experiences affect offspring.^20^ Within TRAILS-NEXT, parent-child interactions were studied during routine vaccinations at 3, 11-12, and 45-48 months, focusing on parental cognitions, emotions, and behaviors before, during, and after vaccination through questionnaires and video observations. Between October 1, 2020, and March 7, 2025, 295 infants aged 3 months, 300 infants aged 11-12 months, and 167 toddlers aged 45-48 months were included in the sample. The current population of participants consisted of 92% European ethnicity with the remaining participants consisting of Surinam, Dutch Antilles, Indonesia, Morocco, Turkey, or another non-European ethnicity. Moreover, the population mostly consisted of participants with a high educational level. Missing values were found for infants aged 11-12 months for educational level and ethnicity (5% and 10%, respectively) and for toddlers aged 45-48 months (26% and 71%, respectively). The participation rate was 77%, with the child already being vaccinated or the parents being too busy as the most mentioned reasons for nonparticipation; only 27 parents (2%) declined participation due to not wanting to vaccinate their child. Table I displays the distribution of sex, ethnicity, educational level, birth order, mode of participation, and number of participants who answered the respective questionnaires. Our sample mostly included mothers (n = 762 [79%]) with 367 (48%) of the children being girls and their first child (n = 405 [53%]) or second child (n = 288 [38%]).

**Table I:** Sample characteristics

Characteristics	3 Months (n = 295)	11-12 Months (n = 300)	45-48 Months (n = 167)
Parent participant (sex): Mother∗	234 (79)	232 (77)	134 (80)
Ethnicity: European∗	270 (92)	250 (93)	48 (91)
Educational level∗			
High	211 (71)	200 (70)	81 (66)
Middle	79 (27)	82 (29)	40 (33)
Low	5 (2)	4 (1)	2 (1)
Child sex: Girl∗	135 (46)	143 (48)	89 (53)
Birth order∗			
First child	143 (48)	159 (53)	103 (62)
Second child	121 (41)	113 (38)	54 (32)
Third child	28 (9)	27 (9)	10 (6)
Fourth child	2 (1)	1 (1)	0
Mode of participation: Video∗	210 (71)	210 (70)	110 (66)
Symptoms in the week preceding vaccination	1 (0-1)	1 (0-2)	0 (0-1)
Parental expectations before vaccination	2 (1-3)	2 (1-3)	2 (0-2)
Parental state anxiety before vaccination	9 (7-11) n = 227	9 (7-10) n = 232	9 (7-11) n = 119
Parental pain ratings directly after vaccination	7 (5-7) n = 232	6 (4-7) n = 240	4 (2-6) n = 121
Parent-reported symptoms in the week after vaccination	2 (1-3)	3 (1-4)	1 (0-3)

Values are number (%) or median (IQR).

∗ All percentages are determined based on nonmissing values.

### Collection Procedure

Data collection took place during 3 routine Dutch vaccination visits at 3, 11-12, and 45-48 months. Younger children received 2 injections with combined vaccines against diphtheria, pertussis, tetanus, and polio; haemophiles influenzae type B; hepatitis B; and pneumococcal bacteria, and older children receiving 1 diphtheria, pertussis, and tetanus injection.^21^ TRAILS-NEXT parents were contacted 4 weeks before vaccination, informed about the study, and asked for consent for video recording and questionnaires. Those declining video recording were invited to complete questionnaires by phone. Exclusion criteria included severe illness, deviations from the regular vaccination schedule, prematurity, disability, or living abroad. For video participants, researchers accompanied parents to youth healthcare centers, which are public health facilities focused on preventive care, routine developmental screenings, and vaccinations for children. Ten minutes before vaccination, parents completed the State-Trait Anxiety Inventory 6-item short form and questionnaires on prior symptoms and expected side effects; nonvideo participants completed these metrics by phone the day before. After vaccination, parents rated their child's pain (Numeric Rating Scale). Seven days later, parents reported on the type and severity of symptoms experienced by their child, and their own behaviors in response to these symptoms by phone. Afterward, participants received a gift card.

## Measures

### Parental State Anxiety

Parental state anxiety shortly before the vaccination of their children was measured using the State-Trait Anxiety Inventory - 6-item short form, a validated short form of the Spielberger State-Trait Anxiety Inventory consisting of 6 items.^22^ Items were “I feel calm,” “I am relaxed,” “I feel content,” “I am worried,” “I feel tense,” and “I am confused.” Items were scored on a 4-point Likert scale, ranging from 1 (not at all) to 4 (very much so); 3 items were reverse coded. The total score ranged from 6 to 24, with higher scores indicating greater anxiety.

### Parental Expectations of Vaccination Side Effects

General parental expectations of side effects were first assessed by the following question: “How likely do you think it is that your child will experience side effects?” with an answering scale of 1 (very unlikely) to 5 (very likely). Subsequently, parents were asked to select the symptoms they expected to occur in the week after vaccination from the following age-appropriate list: decreased appetite, tiredness or trouble sleeping, difficulty breathing, coughing, runny nose or a cold, vomiting, fever, diarrhea, crying more than usual, redness/bruise or itching and/or pain, hardening or swelling around the inoculation, large swelling of the vaccinated limb, and other (at 3 and 11-12 months of age); or stomach ache or an uncomfortable feeling in the stomach; headache or tense/painful neck and shoulders; difficulty breathing or rapid breathing; pain in the joints, legs, or arms; urination problems or frequent urination; nausea or vomiting; throat pain; coughing; runny nose or a cold; earache; increased temperature or fever; decreased appetite; tiredness; diarrhea; redness/bruise or itching and/or pain; hardening or swelling around the inoculation; large swelling of the vaccinated limb; and other (45-48 months of age). This questionnaire was constructed for this study and based on the most prevalent physical symptoms for young children reported in previous research and the most prevalent side effects reported for the given vaccines.^4^^,^^5^^,^^23^^,^^24^

### Parental Ratings of Child Vaccination Pain

Directly after the vaccination, parents were asked how much pain they thought their child had experienced during the vaccination, using the 0-10 Numeric Rating Scale where 0 is equal to “no pain” and 10 to “the worst pain imaginable.”^25^

### Parental Ratings of Physical Symptoms Post Vaccination

Seven days after the vaccination procedure, parents were asked to complete a questionnaire by telephone regarding the actual side effects that they observed in their child during the week after vaccination. Parents were first asked: “To what extent has your child suffered from physical symptoms in the past week?” with an answering scale of 1 (not at all) to 4 (very much). Afterward, all parents were asked to select which specific symptoms their child had experienced, using the same age-appropriate symptom list as before vaccination.

### Health Behaviors

Parents were asked about several potential behaviors in the 7 days following vaccination, with binary items (yes/no) and follow-up questions if they indicated the behavior applied to them. These behaviors included (1) asking advice from (1a) a near one/nonprofessional, (1b) the youth healthcare center (if yes: in person or by phone), or (1c) the general practitioner; (2) seeking for information in (2a) the vaccination information leaflet provided by the youth healthcare center, or (2b) online (if yes: before or after vaccination, which websites); (3) temperature measuring (if yes: how often and highest temperature measured); (4) providing medication (if yes: which medication[s]); and (5) keeping their child at home from daycare or school. This questionnaire was specifically developed by our research team for use in the current study, because no suitable existing instrument was available to address our specific research aims and population.

### Statistical Analyses

All included analyses in this study were preregistered before the start of the analyses (https://osf.io/4pxfz). In discordance with the preregistration, we randomly selected 1 child per parent in each age group before analyses, because some parents participated with multiple children belonging to the same age group. Statistical analyses were conducted using SPSS Statistics 28 (SPSS, Inc). We applied Holm-Bonferroni correction for multiple comparisons. All analyses were performed for each age group separately, because individual parents and their children were often included in multiple age groups, and because we were interested in exploring differences in parental cognitions, emotions and behaviors based on the age of the child.

To test parental prevaccination factors in relation to side effects, multivariable linear regression analyses were performed with both parent-reported total number of expectations of side effects and parental state anxiety as independent variables, and parent-reported child pain immediately after vaccination (hypothesis 1) or total number of parent-reported symptoms experienced by the child in the week after vaccination (hypothesis 2) as dependent variables. The relation between specific expected symptoms before vaccination and specific observed symptoms in the week after vaccination (hypothesis 3) was assessed using Spearman's correlation and visualized using heatmaps.

To explore health behaviors in the week after vaccination in relation to observed side effects, we divided the parents into 3 groups based on the parent-reported level of physical symptom burden in the child in the week after vaccination, reflecting “not at all” (score of 1), “somewhat” (score of 2), and “(very) much” (score of 3 or 4). We also explored these health behaviors in relation to parents’ perceived likelihood that their child would experience vaccination side effects. Parents were divided into 3 groups based on their answer, reflecting “(very) unlikely,” “neutral,” and “(very) likely.” We defined the relevancy of observed and expected symptoms for health behaviors as a consistent increase in the frequency of these behaviors.

### Covariates

To adjust for already existing symptoms, the number of pre-existing symptoms reported by parents in the week before vaccination, excluding local skin reactions, and mode of participation (video vs questionnaire) were added as a covariate. We also included child sex, because it has been linked to differences in perceived side effects and help seeking,^13^^,^^15^ and parent sex, given that mothers often hold stronger vaccination opinions.^26^ Birth order was included due to its association with parental help seeking.^27^ Child and parent sex and child age were recorded at consent; birth order was based on parental report.

## Results

Generally, parents reported few side effect expectations, low state anxiety, and few symptoms before vaccination in each age group. Parental reports of pain directly after vaccination and symptoms in the week after vaccination were found to be higher for younger children. Missing values for parental state anxiety before vaccination and parental pain ratings directly after vaccination ranged between 20% and 29%. These missing values almost exclusively occurred in the group that completed the questionnaires online via text message as opposed to the group participating with video observations (Table I). When including mode of participation as a covariate, we found no differences in results between parental prevaccination state anxiety and acute pain ratings collected via text messages or in person (Tables II and III).

**Table II:** Associations between parental prevaccination state anxiety and total number of expectations and acute pain report of their child

Characteristics	Children aged 3 months		Children aged 11-12 months		Children aged 45-48 months	
	Standardized Beta	95% CI	Standardized Beta	95% CI	Standardized Beta	95% CI
Parental state anxiety	0.254∗	[0.070 to 0.245]	0.117	[−0.015 to 0.190]	0.246∗	[0.063 to 0.426]
Parental expectations	−0.016	[−0.206 to 0.166]	0.138†	[0.004 to 0.375]	0.122	[−0.126 to 0.580]
Sex of the parent	−0.093	[−0.958 to 0.182]	−0.058	[−0.793 to 0.306]	−0.110	[−1.698 to 0.451]
Sex of the child	−0.019	[−0.508 to 0.379]	−0.051	[−0.651 to 0.284]	0.149	[−0.145 to 1.524]
Birth order	0.015	[−0.294 to 0.366]	0.151†	[0.056 to 0.806]	0.124	[−0.240 to 1.186]
Symptoms in the week preceding vaccination	−0.025	[−0.260 to 0.179]	0.049	[−0.101 to 0.209]	0.021	[−0.360 to 0.453]
Video vs questionnaire only participation	0.042	[−0.431 to 0.820]	0.115	[−0.070 to 1.181]	−0.001	[−1.109 to 1.092]

∗ Significant at the 0.01 level.

† Significant at the 0.05 level.

**Table III:** Associations between parental prevaccination state anxiety and total number of expectations and total number of reported symptoms in the week after vaccination

Characteristics	Children aged 3 months		Children aged 11-12 months		Children aged 45-48 months	
	Standardized Beta	95% CI	Standardized Beta	95% CI	Standardized Beta	95% CI
Parental state anxiety	0.104	[−0.021 to 0.148]	−0.103	[−0.196 to 0.029]	−0.072	[−0.215 to 0.097]
Parental expectations	0.112	[−0.036 to 0.319]	0.009	[−0.190 to 0.216]	0.190	[−0.008 to 0.595]
Sex of the parent	0.009	[−0.514 to 0.591]	0.032	[−0.449 to 0.736]	0.071	[−0.592 to 1.275]
Sex of the child	−0.033	[−0.536 to 0.322]	−0.009	[−0.544 to 0.472]	0.039	[−0.564 to 0.865]
Birth order	0.029	[−0.254 to 0.391]	−0.046	[−0.546 to 0.265]	−0.059	[−0.802 to 0.424]
Symptoms in the week preceding vaccination	0.154∗	[0.036 to 0.457]	−0.001	[−0.169 to 0.168]	−0.059	[−0.461 to 0.243]
Video vs questionnaire only participation	−0.069	[−0.844 to 0.265]	0.069	[−0.304 to 0.970]	−0.099	[−1.369 to 0.432]

∗ Significant at the 0.05 level.

### Parental Prevaccination State Anxiety and Total Number of Expectations and Parental Pain Report in Their Child Directly after Vaccination

Table II displays the results of the linear regression analysis testing whether parental prevaccination state anxiety and total number of side effect expectations predict parent-reported acute pain in their child during vaccination. Parental prevaccination state anxiety was associated with higher pain reports at 3 months (β = 0.254; *P* ≤ .001) and 45-48 months (β = 0.246; *P* = .009), with small to medium effect sizes. After correcting for multiple testing however, the association at 45-48 months did not remain significant. The association between the total number of parental expectations of side effects and acute pain was significant at 11-12 months (β = 0.138; *P* = .046), with a small effect size. After correcting for multiple testing, this result did not remain significant. At 3 months or 45-48 months, we found no significant associations.

### Parental Prevaccination State Anxiety and Total Number of Expectations and Total Number of Reported Symptoms in Their Child 1 Week after Vaccination

Table III displays the linear regression analysis testing whether parental prevaccination state anxiety and total number of expectations predict the total number of symptoms 1 week after vaccination. No significant associations were found between parental expectations or parental prevaccination state anxiety and reported symptoms 1 week after vaccination at 3, 11-12, or 45-48 months.

### Correlations between Expected and Observed Symptoms 1 Week after Vaccination

The Spearman's rank-order correlation analyses were transformed into respective heatmaps to visualize the overall correlation between parental expectations of specific symptoms and observed symptoms in their child 1 week after vaccination. Analyses were restricted to symptoms that were expected at least 10 times. The respective heatmaps are shown in (Figures 2-4 in Appendix A; available at www.jpeds.com).

At 3 months, the strongest correlations appeared on the diagonal of the heatmap, indicating higher within-symptom than between-symptom correlations. These within-symptom correlations ranged from ρ = 0.151 to ρ = 0.259. At 11-12 months, the heatmap displayed fewer significant correlations between specific symptom expectations and observations than at 3 months, ranging from ρ = 0.115 to ρ = 0.175. Last, at 45-48 months, the heatmap displayed fewer significant correlations within specific symptom expectations and observations, but more between-symptom correlations, ranging from ρ = 0.163 to ρ = 0.236.

### Exploratory Analysis: Distribution of Parental Health Behaviors

Figure displays the health behaviors of parents in each age group. Parents of 3-month-old children reported the most health behaviors overall, declining with each older age group. Measuring the temperature of the child and providing medication were the most frequent behaviors, reported by more than one-half of parents with infants, decreasing to 20% at 45-48 months. Seeking information was reported by a minority of the parents, more often in the vaccination leaflet than online. Asking advice and keeping the child home were not frequently performed.

**Figure:**
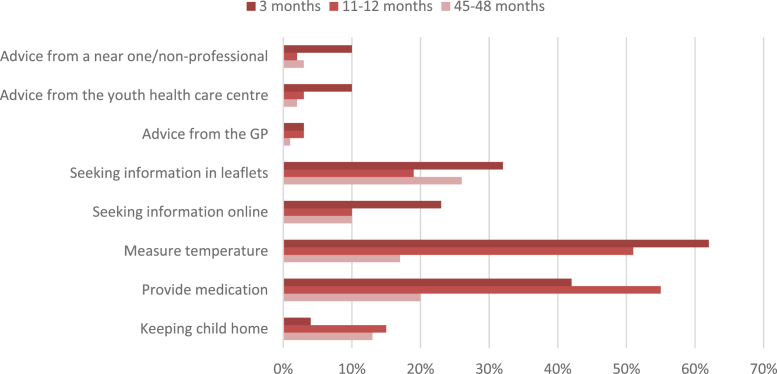
Parent-reported health behaviors.

### Exploratory Analysis: Parental Health Behaviors in Relation to Observed Symptom Suffering 1 Week after Vaccination and Expected Symptom Likelihood

Subsequently, we explored whether behavioral responses were more frequent in parents who observed more symptom suffering in their child (Figure 5 A-C, Appendix B; available at www.jpeds.com). A clear positive relationship was evident between the degree of suffering observed and the frequency of resulting health behaviors, which became more prominent with increasing age. Parents who reported (very) much suffering, measured temperature 2 times more often at 3 months, 3 times more often at 11-12 months, and 15 times more often at 45-48 months compared with parents who reported no suffering. Providing medication occurred 3 times more often at 3 months, 4 times more often at 11-12 months, and 7 times more often at 45-48 months, compared with parents who reported no suffering.

Finally, we divided parents into groups based on their expected likelihood that symptoms would appear. We identified no consistent patterns of health behaviors in the groups differing in side effect expectations across ages (Figure 5 D-F, Appendix B; available at www.jpeds.com). At 3 months, only measuring temperature clearly increased with higher symptom expectations. At 11 months, consistently increasing frequencies were found for measuring temperature, but differences between groups were small. At 45-48 months, the only consistent increase in relation to symptom expectations was found for seeking information in leaflets and providing medication.

## Discussion

This study aimed to investigate whether parental expectations of vaccination side effects and parental prevaccination state anxiety were associated with parent-reported pain in their child immediately after vaccination and observed symptoms and health behaviors in the week after vaccination. We found that parental prevaccination state anxiety was significantly associated with higher parental reports of their child's acute pain during vaccination at 3 months. Parental side effect expectations were not associated with parental reports of acute pain, and neither parental prevaccination state anxiety nor expectations were associated with the total number of symptoms parents reported 1 week after vaccination. Analyses related to specific symptoms suggested that observed symptoms were related to symptom expectations at 3 months, but not at older ages. In each age group, measuring temperature and providing medication were the most frequently reported health behaviors by parents. The frequency of these behaviors was associated with the observed suffering in their children across all age groups, but less so to symptom expectations.

Our findings on the positive associations between parental prevaccination state anxiety and reported acute vaccination pain are in line with previous research on the correlations between parental prevaccination anxiety and pain perception in parental reports of pain during vaccination in children younger than 24 months.^17^ An explanation for the difference in associations between 3 and 11-12 months may lie in parents' increased experience with the vaccination process at the older age, which might have influenced their interpretation of the pain-related distress behaviors they observed in their child. Whether parental perception of acute pain reflects a heightened pain experience in their child remains difficult to study, given the reliance on parents for reporting symptoms in young children. A study in preschoolers suggested that parental prevaccination anxiety influences the child's pain experience via an increase in the child's procedural anxiety.^9^ Ample research has suggested that children are sensitive to their parents' behavior, which may result in the child learning to be anxious for vaccination, potentially increasing perceived pain during vaccination.^28^ Our initial analysis also revealed significant associations at 45-48 months, which did not remain significant after multiple testing and should thus be interpreted cautiously. At this age, the child's increased ability to communicate symptoms, combined with potential fear and anxiety, may have contributed to higher parental pain reports.^9^^,^^29^ Besides parental prevaccination state anxiety, other factors not investigated in the current study might also contribute to higher acute pain reports by the parent. One example is parents' own experiences with vaccinations and acute pain or parental worry of how their child will experience vaccination.^30^^,^^31^

We found no associations between the total number of parental side effect expectations and prevaccination state anxiety on the parental report total number of symptoms 1 week after vaccination. This finding contrasts with the only other study on parental expectations, which found a significant association between side effect expectations and parental report of symptoms 3 days and 1 month after vaccination.^12^ This discrepancy may stem from methodological differences. The previous study related whether parents expected symptoms (yes/no) to the appearance of at least 1 nonspecific symptom 3 days and 1 month later, whereas we related the total number of symptoms parents expected to the total number of symptoms parents observed in their child 1 week after vaccination. Another explanation is differences between the samples in their knowledge of the likelihood and nature of side effects. The sample in the previous study received the influenza vaccine, which may have influenced both expectations and observed symptoms.

Although we found no general association between the total number of expected and observed symptoms, our analyses suggest that specific symptom expectations are associated with specific observed symptoms in infants. When examining the correlations for children aged 3 months, we found that parents who reported specific expectations tended to observe those same symptoms 1 week after vaccination. This result is in line with our observation that parents of children aged 3 months were more likely to ask advice, seek information, and measure their child's temperature compared with parents of older children. Previous research on the nocebo effect highlights that higher symptom expectations can lead to paying increased attention to potential symptoms related to those expectations.^16^ This mechanism may play a role in the parental observation of symptoms in children aged 3 months, because infants are unable to verbalize or indicate their own symptoms, potentially leading to parents observing the specific symptom in line with their expectations. The associations between observed and expected symptoms were not evident at older ages, in line with decreased total health behaviors reported by parents in our sample, possibly due to developmental changes in parenting, as well as the communicative abilities of children. When analyzing parental health behaviors in response to symptom burden, however, we found an increase in the health behaviors performed by parents who reported a higher symptom burden in their older child. The enhanced communicative skills of older children may have resulted in the increase in health behaviors performed by parents, such as providing medication or measuring temperature at this age.^29^ Some age-specific associations with health behaviors were also found for symptom expectations, but patterns were not consistent and differences were often small.

## Strengths and Limitations

This study contributes to the limited evidence investigating the influence of parental factors associated with observing acute vaccination pain and side effects in their children. Moreover, to our knowledge, we are the first to explore various health behaviors in parents in relation to their side effect expectations and observed symptom burden in their child after vaccination. A major strength of the study is the high participation rate of 77% with a final sample of more than 750 measurements, including children at various ages during their routine vaccinations. Data were collected shortly before vaccination, immediately after vaccination, and 1 week after vaccination, allowing parents to complete the questionnaires at key points in the vaccination process.

Several limitations should be considered for this study. First, some of the parents completed the questionnaires in the waiting room, while others responded to a text message with a link to an online questionnaire. Because the latter group included parents who only completed questionnaires online, due to their own preferences or operational constraints within the research team, this group might systematically differ from the group consenting to be video recorded. Although this factor might be considered a limitation, the fact that parents not consenting to video recordings or parents not filmed for practical reasons were offered an alternative procedure to participate increases the external validity of the study. In the questionnaire-only group, more missing values occurred due to technical problems with the text message link. To account for possible differences, all our analyses were adjusted for mode of participation (video or questionnaire), with no major influence on the results.

Second, the questionnaires assessing parental expectations, symptoms in the week before and after vaccination, and parental health behaviors in the week after vaccination were specifically designed for this study. Because we were among the first to examine these concepts, validated questionnaires were not available for this study.

Third, the TRAILS cohort may not fully represent families who are less likely to participate in longitudinal research or have different views on science, healthcare, or vaccination. Our sample's predominance of European ethnicity and high vaccination rates may reduce generalizability to more diverse populations or those with different vaccination-related anxieties and decisions. Future studies in varied demographic, cultural, and ethnic contexts are warranted to determine whether these findings apply more broadly.

Last, the child's health status was not included in our analyses, although this factor might have influenced parental prevaccination state anxiety and side effect expectations. However, we only included children vaccinated at the youth healthcare center, which likely excluded children with severe illnesses; these children are vaccinated in hospital settings in the Netherlands.

## Implications

Our results highlight that, although parents did not report high levels of prevaccination state anxiety, it nevertheless was associated with an increase in reports of acute pain in their young child. To further reduce parental anxiety, especially during 3-month vaccinations, parents should be well informed and properly guided throughout the vaccination process. In this context, youth healthcare centers should be more aware of the anxiety parents may have regarding vaccination and offer strategies to better manage this. Presently, information on vaccination primarily focuses on the importance of vaccination and the potential side effects parents may expect, overlooking parental anxiety. Future research could focus on better understanding factors influencing parental state anxiety as the child grows older and is more able to report on pain and symptoms. In addition, various child factors remain unexplored in the current study, which may influence parental acute pain reporting, such as child pain-related behaviors or the temperament of the child.^30^^,^^31^

Although vaccination side effects are generally mild and short lived, parents still frequently report performing health behaviors.^1^, ^2^, ^3^ The influence of parental health behaviors, explored in this study, on their children's vaccination anxiety and symptom experience remains poorly understood. Further research is needed to better understand the influence of parental emotions and cognitions on postvaccination health, especially in younger children, where parents appear to engage in most of these behaviors. As children observe their parents' responses to symptoms, such as temperature monitoring and giving medication, they may learn that these behaviors are important for managing illness or discomfort.^32^ Whether children adopt these health behaviors from their parents through social learning may shape their symptom perception and illness behavior in the next generation. Our population consisted primarily of western ethnicity; as such, further research is needed to investigate differences in ethnicity on the results. Although we examined parental health behaviors after vaccination, future studies could focus on the health behaviors of the child before and during the vaccination itself to assess how parental behaviors shape the future experiences with healthcare use and procedural pain for their children. Using multiple research methods will be essential to further elucidate the cognitions, emotions, and behaviors surrounding health and illness in both parent and child.

## CRediT authorship contribution statement

**Brian A. Jorge:** Writing – original draft, Resources, Methodology, Investigation, Formal analysis, Data curation, Conceptualization. **Sterre van der Ziel:** Writing – review & editing, Supervision, Resources, Methodology, Investigation, Data curation, Conceptualization. **Elske Hogendoorn:** Writing – review & editing, Resources, Methodology, Investigation, Data curation. **Michel J. van Vliet:** Writing – review & editing, Supervision. **Judith G.M. Rosmalen:** Writing – review & editing, Supervision, Project administration, Methodology, Funding acquisition, Conceptualization.

## Declaration of Competing Interest

This research did not receive any specific grant from funding agencies in the public, commercial, or not-for-profit sectors.

The authors declare no conflicts of interest.

## Supplementary Data

Appendix A and B

Data Statement
