# Supplementary material for: Parental Expectations, Anxiety, and Health Behaviors in Routine Childhood Vaccination: Associations with Child Pain and Symptom Experiences
Source: J Pediatr Clin Pract. 2026 Jul 1;21:200227. doi: 10.1016/j.jpedcp.2026.200227 (PMC13427399; doi:10.1016/j.jpedcp.2026.200227)

**Appendix A**

**Figure 2.** Correlations between parental expectations and reported symptoms one week after vaccination at age 3 months.

| **3 months**  **Reported symptoms**  **Expectations** | 1 (n=36) | 2 (n=162) | 3 (n=47) | 5 (n=124) | 5 (n=27) | 6 (n=154) | 7 (n=74) |  |  |  |  |
| --- | --- | --- | --- | --- | --- | --- | --- | --- | --- | --- | --- |
| 1. Decreased appetite (n=38) | 0.104 | 0.084 | 0.054 | 0.042 | 0.053 | 0.003 | .151^**^ |  |    \|  \| \| --- \| |  |  |
| 2. Tiredness / trouble sleeping (n=145) | .151^**^ | .155^**^ | -0.039 | 0.069 | 0.064 | -0.050 | -0.021 |  |  |  |  |
| 3. Coughing / runny nose (n=13) | -0.030 | 0.029 | 0.087 | -0.082 | -0.011 | -0.026 | -0.048 |  |  |  |  |
| 4. Fever (n=161) | 0.070 | 0.049 | -0.049 | .170^**^ | 0.030 | 0.081 | .167^**^ |  |  |  |  |
| 5.  Diarrhea (n=7) | 0.010 | 0.052 | -0.007 | 0.048 | .259^**^ | 0.015 | 0.064 |  |  |  |  |
| 6. Crying more than usual (n=146) | 0.045 | 0.038 | 0.051 | 0.022 | -0.009 | 0.038 | 0.037 |  |  |  |  |
| 7. Inoculation site symptoms (n=79) | -0.038 | 0.086 | -0.033 | 0.074 | 0.020 | 0.042 | .215^**^ |  |  |  |  |
| Darker colors indicate a higher correlation with its respective significant correlation coefficient. | | | | | | | | |  |  |  |
| **Correlation is significant at the 0.01 level. | | | | | | | | |  |  |  |
|  | | | | | | | | |  |  |  |

**Figure 3**. Correlations between parental expectations and reported symptoms one week after vaccination at age 11-12 months.

| \| **11-12 months**  **Expectations** \| \| --- \| | 1 (n=60) | 2 (n=154) | 3 (n=89) | 4 (n=136) | 5 (n=19) | 6 (n=29) | 7 (n=143) | 8 (n=92) |  |  |  |
| --- | --- | --- | --- | --- | --- | --- | --- | --- | --- | --- | --- | --- |
| 1. Decreased appetite (n=23) | .169^**^ | 0.005 | .115^*^ | 0.014 | 0.028 | 0.033 | 0.051 | -0.029 |  |  |  |
| 2. Tiredness / trouble sleeping (n=134) | 0.020  **Reported symptoms** | 0.110 | -0.026 | -0.024 | 0.042 | 0.024 | 0.069 | 0.013 |    \|  \| \| --- \| |  |  |
| 3. Coughing, runny nose (n=18) | -0.021 | -.175^**^ | -0.010 | -0.061 | -0.008 | 0.035 | -0.073 | 0.015 |  |  |  |
| 4. Fever (n=175) | 0.085 | -.133^*^ | 0.046 | .118^*^ | 0.025 | -0.067 | -0.006 | 0.049 |  |  |  |
| 5. Vomiting (n=2) | 0.061 | -0.002 | -0.053 | 0.090 | -0.021 | -0.027 | -0.078 | -0.054 |  |  |  |
| 6. Diarrhea (n=9) | 0.010 | -0.102 | 0.014 | -.121^*^ | 0.034 | 0.009 | -0.011 | -0.032 |  |  |  |
| 7. Crying more than usual (n=142) | 0.010 | -0.079 | -0.002 | -0.005 | 0.082 | 0.006 | .124^*^ | -0.022 |  |  |  |
| 8. Inoculation site symptoms (n=91) | 0.033 | -0.054 | 0.048 | -0.062 | -0.052 | 0.054 | -0.078 | 0.033 |  |  |  |
| *Correlation is significant at the 0.05 level. | | | | | | | | | | |  |
| **Correlation is significant at the 0.01 level. | | | | | | | | | | |  |

**Figure 4.** Correlations between parental expectations and reported symptoms one week after vaccination at age 45-48 months.

| \| **45-48 months**  **Expectations**  **Reported symptoms** \| \| --- \| | 1 (n=18) | 2 (n=21) | 3 (n=9) | 4 (n=41) | 5 (n=45) | 6 (n=25) | 7 (n=54) | 8 (n=10) | 9 (n=83) |  |  |  | |  |
| --- | --- | --- | --- | --- | --- | --- | --- | --- | --- | --- | --- | --- | --- | --- | --- |
| 1. Stomach ache (n=2) | -0.032 | -0.035 | -0.019 | -0.055 | -0.058 | -0.037 | -0.066 | -0.023 | -0.091 |  |  |  | |  |
| 2. Pain in the joints, legs or arms (n=25) | -0.043 | .197^*^ | 0.039 | 0.084 | 0.109 | .171^*^ | 0.101 | 0.101 | .164^*^ |    \|  \| \| --- \| |  |  | |  |
| 3. Nausea or vomiting (n=5) | -0.046 | -0.049 | -0.028 | -0.078 | -0.082 | -0.053 | -0.094 | .163^*^ | 0.030 |  |  |  | |  |
| 4. Throat pain, coughing, runny nose (n=8) | 0.039 | 0.028 | 0.125 | 0.100 | 0.087 | 0.110 | 0.120 | -0.047 | 0.099 |  |  |  | |  |
| 5. Fever (n=105) | 0.003 | -0.042 | 0.024 | 0.012 | 0.111 | .196^*^ | -0.012 | -0.104 | 0.141 |  |  |  | |  |
| 6. Decreased appetite (n=20) | 0.133 | 0.040 | -0.057 | -0.008 | -0.022 | 0.092 | 0.036 | .236^**^ | 0.020 |  |  |  | |  |
| 7. Tiredness (n=74) | 0.105 | -0.064 | 0.013 | 0.030 | 0.086 | 0.115 | 0.139 | 0.027 | -0.021 |  |  |  | |  |
| 8. Diarrhea (n=3) | -0.039 | -0.042 | -0.024 | -0.067 | 0.039 | -0.046 | 0.021 | -0.028 | -0.020 |  |  |  | |  |
| 9. Inoculation site symptoms (n=69) | 0.082 | 0.047 | 0.026 | 0.004 | 0.097 | 0.059 | -0.072 | 0.108 | -0.010 |  |  |  | |  |
| *Correlation is significant at the 0.05 level. | | | | | | | | | | | | |  | |
| **Correlation is significant at the 0.01 level. | | | | | | | | | | | | |  | |

**Appendix B**

**Figure 5.** Health behaviors in relation to symptom burden (A,B,C) and expected likelihood of symptoms (D,E,F) at 3, 11-12 and 45-48 months


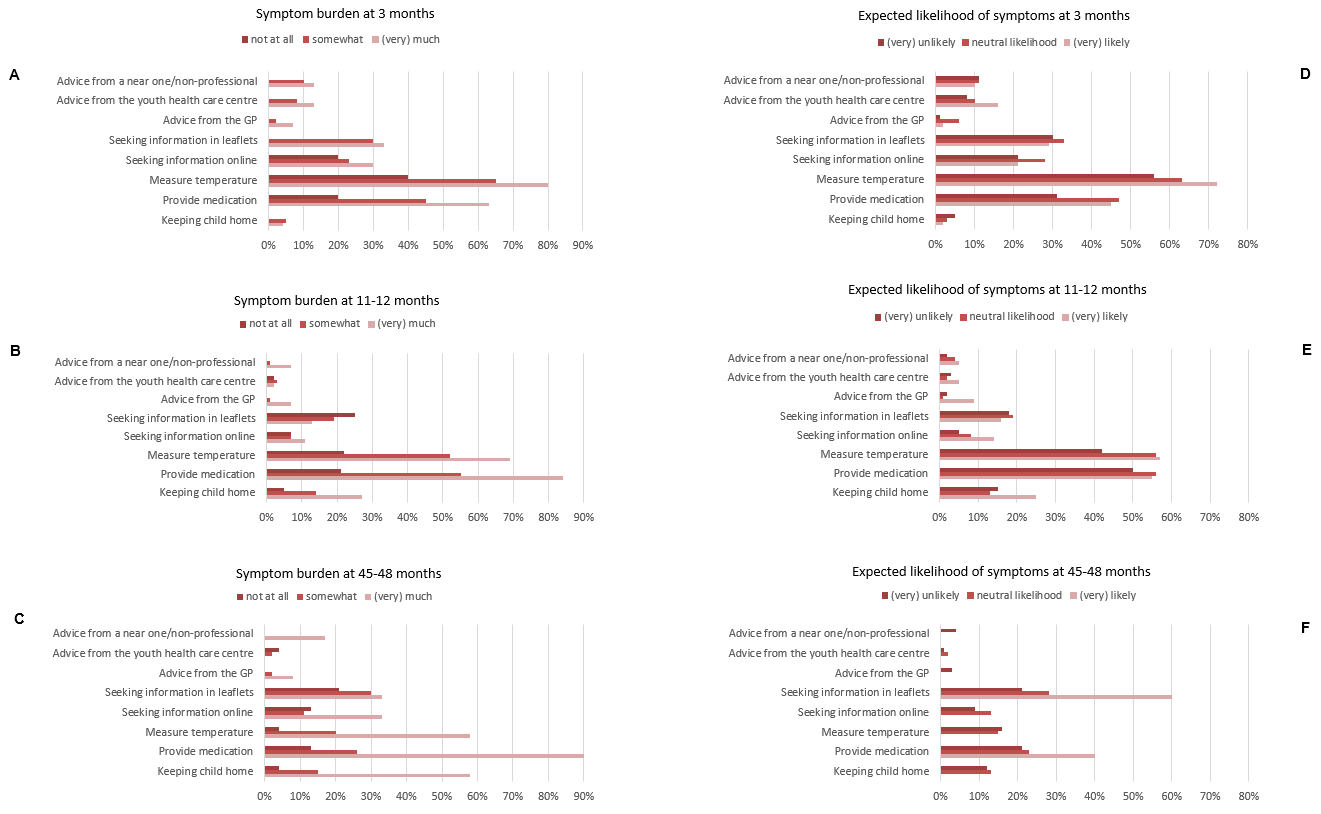

Supplement: Appendix A and B [file mmc1.docx]
