# Supplementary material for: Parental Expectations, Anxiety, and Health Behaviors in Routine Childhood Vaccination: Associations with Child Pain and Symptom Experiences
Source: J Pediatr Clin Pract. 2026 Jul 1;21:200227. doi: 10.1016/j.jpedcp.2026.200227 (PMC13427399; doi:10.1016/j.jpedcp.2026.200227)
Supplement: Data Statement [file mmc2.docx]

Data Statement

Due to the sensitivity of the data and the restrictions in informed consent, the data will not be stored at a public repository. The project data and documentation will be stored at a repository at the UMCG, which ensures the security of the data and back-up. UMCG pursues a FAIR data policy for research conducted in the institute. To make the data findable and accessible for researchers inside and outside the institute, a description of the TRAILS data (metadata) is included in the UMCG Research Data catalogue (<https://umcgresearchdatacatalogue.nl/UMCG/catalogue/all/collections/TRAILS>). This catalogue will be in sync with relevant (inter)national catalogues, such as BBMRI and NARCIS. A data access committee has been put in place that will review requests and assure accessibility of the data, provided there is a lawful basis for sharing the data. This access committee can be reached via [trails@umcg.nl](mailto:trails@umcg.nl).
